# Dry Eye Management: Targeting the Ocular Surface Microenvironment

**DOI:** 10.3390/ijms18071398

**Published:** 2017-06-29

**Authors:** Xiaobo Zhang, Vimalin Jeyalatha M, Yangluowa Qu, Xin He, Shangkun Ou, Jinghua Bu, Changkai Jia, Junqi Wang, Han Wu, Zuguo Liu, Wei Li

**Affiliations:** 1 Eye Institute of Xiamen University, Xiamen 361102, China; xiaoboz@xmu.edu.cn (X.Z.); mvimalin@gmail.com (V.J.M.); quyangluowa@126.com (Y.Q.); hexin19921016@163.com (X.H.); Shangkun_Ou@126.com (S.O.); bujinghua2009@163.com (J.B.); ck_jia@126.com (C.J.); blacwing@163.com (J.W.); xywpwh@163.com (H.W.); zuguoliu@xmu.edu.cn (Z.L.); 2 Medical College of Xiamen University, Xiamen 361102, China; 3 Xiamen University affiliated Xiamen Eye Center, Xiamen 361102, China; 4 Fujian Provincial Key Laboratory of Ophthalmology and Visual Science, Xiamen 361102, China

**Keywords:** dry eye, ocular surface, microenvironment, homeostasis

## Abstract

Dry eye can damage the ocular surface and result in mild corneal epithelial defect to blinding corneal pannus formation and squamous metaplasia. Significant progress in the treatment of dry eye has been made in the last two decades; progressing from lubricating and hydrating the ocular surface with artificial tear to stimulating tear secretion; anti-inflammation and immune regulation. With the increase in knowledge regarding the pathophysiology of dry eye, we propose in this review the concept of ocular surface microenvironment. Various components of the microenvironment contribute to the homeostasis of ocular surface. Compromise in one or more components can result in homeostasis disruption of ocular surface leading to dry eye disease. Complete evaluation of the microenvironment component changes in dry eye patients will not only lead to appropriate diagnosis, but also guide in timely and effective clinical management. Successful treatment of dry eye should be aimed to restore the homeostasis of the ocular surface microenvironment.

## 1. Introduction

### 1.1. Current Strategy on Dry Eye Management

Dry eye has become the most common ocular surface disease throughout the world. In 2007, the International Dry Eye Workshop (DEWS) defined dry eye as a multifactorial disease of the tear and ocular surface that results in symptoms of discomfort, visual disturbance, and tear film instability with potential damage to the ocular surface [1]. The well accepted theory on the pathophysiology of dry eye is the concept of lacrimal functional unit (LFU), which was proposed by Stern ME et al. about two decades ago [2]. LFU comprises the ocular surface (cornea, conjunctiva, and meibomian glands), the main and accessory lacrimal glands, and the neural network that connects them; it controls secretion of the tear film in a regulated fashion. LFU concept was broadly applied to study the physiology and pathophysiology of tear secretion. Dry eye disease (DED) is recognized as a disturbance of the LFU [1].

Current strategy on dry eye treatment is also mainly based on the concept of LFU. DEWS has tabulated various approaches for dry eye management which includes the use of artificial tears, anti-inflammatory agents, tetracyclines, puncta plugs, secretogogues, serum, contact lens, systemic immunosuppressives, and surgical interventions [1]. These approaches are aimed to eliminate exacerbating factors, support the tear-producing glands, hydrate the ocular surface, restore normal tear film osmolarity, stabilize the tear film, inhibit inflammation, and eventually restore normal function of LFU. With these approaches, general outcome of DED treatment has significantly improved in the past two decades.

### 1.2. The Concept of Ocular Surface Microenvironment

**Figure 1:**
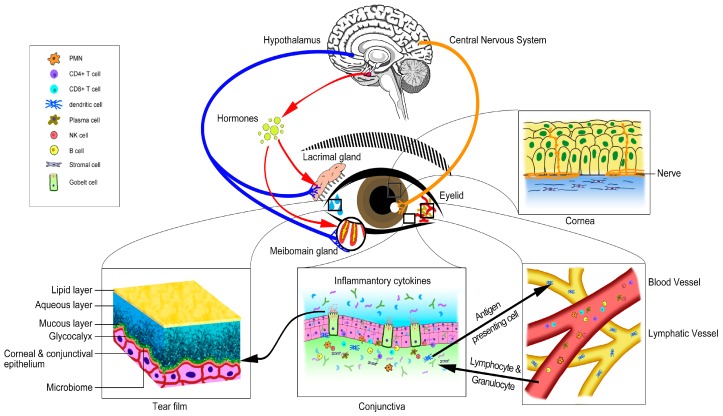
Schematic representation of ocular surface microenvironment components. The cornea, conjunctiva, meibomian glands, lacrimal glands and their neural network as well as other components such as immune cells, matrix, hormones, small molecules and also microbiome interact together to form the ocular surface microenvironment which is pivotal for the healthy ocular surface.

The ocular surface is a delicate and complex system. Rather than cornea, conjunctiva, meibomian glands, lacrimal glands and the neural network, other components such as immune cells, matrix cells, hormones and even microbiome also regulate the homeostasis of the ocular surface. We propose that all these tissues, specific cells, matrix, small molecules and microbiome interact together to form the ocular surface microenvironment (OSM, Figure 1). Normal OSM is pivotal for the healthy ocular surface. Some components of the OSM may not have direct association with the LFU, however, many evidence show that with the change of these components, the homeostasis of the ocular surface can be compromised, eventually leading to the dysfunction of LFU and resulting in dry eye.

Therefore, the foremost objective in the DED management is to restore the OSM to its normal homeostatic state. Complete evaluation of the OSM components will aid in successful restoration of ocular surface homeostasis. In this review, we try to illustrate the components of ocular surface in the angle of OSM, interpret the interaction of different components of OSM, and demonstrate the changes of OSM components during dry eye and how dry eye affects OSM components. We have also discussed the application of OSM concept in the treatment of dry eye.

## 2. The Components of Ocular Surface Microenvironment

### 2.1. Tissue Components of the Ocular Surface Microenvironment

The ocular surface is composed of different tissue components, and all these components work together harmoniously to maintain the integrity and normal function of the ocular surface. Here, we focus on the normal function of different ocular surface tissues and their interaction regarding the maintenance of normal OSM.

#### 2.1.1. Cornea

Cornea play an important role in maintaining the balance of ocular surface microenvironment. Corneal epithelium is instantly exposed to the external environment. They resist the entry of noxious pathogens by maintaining the tight intracellular junctions. The microvilli of the superficial epithelium aids in the anchorage of the tear film. The highly organized and stratified epithelium also produces the o-glycosylated transmembrane mucins contributing to the glycocalyx region. The epithelial cells possess various signaling mechanisms involved in wound healing and also secret enormous proteins such as growth factors, cytokines, etc. [3]. Keratocytes in the corneal stroma are capable of synthesizing collagen and glycosaminoglycans maintain the extracellular matrix. Keratocytes also interact with epithelial cells through secreting signaling pathway ligands. Limbal stromal cells act as niche cells for the limbal epithelial stem cells thus control proliferation and differentiation of limbal stem cells [4]. Meanwhile, the cornea possess unmyelinated nerve endings that generate afferent nerve impulses for the lacrimal functional unit. Corneal endothelium acts as a barrier between the corneal stroma and the aqueous humor in the anterior chamber.

#### 2.1.2. Conjunctiva

Conjunctiva covers two-thirds of the ocular surface from the corneal rim to the lid margin [5]. The epithelium of conjunctiva has the similar barrier function as corneal epithelium; it also plays an important role in mucin secretion and immune defense. The conjunctival epithelium house the mucin producing goblet cells and the dendritic Langerhans cells. Conjunctival goblet cells synthesize and secrete a large family of mucins including MUC1, MUC4, MUC5AC and MUC16 [6,7]. It has been reported that conjunctival goblet cells are surrounded by lymphocytes and dendritic cells and their density has been found to change in certain ocular surface immune/inflammatory conditions and it may be the direct target of cytokines and chemokines [8]. The conjunctiva has resident lymphoid cells, including dendritic cells, natural killer (NK) cells, B-cells and T cells that suppress [8,9,10] or promote [8] immune responses. Conjunctival stroma act as support tissue for the cornea and conjunctiva. Reports also show that the mesenchymal stem/stromal cells (MSCs) reside in the conjunctival stroma involving in tissue repair and modulating excessive immune responses [11,12].

#### 2.1.3. Lacrimal Glands

The lacrimal gland contributes to the normal hemostasis of OSM through secreting the aqueous tear that includes water, electrolytes, protein, and mucus. Sufficient volumes of fluid secretion maintain the moisture content of the ocular surface, keeps the proteins in solublized state, act as light refraction medium between the air and the cornea. The lacrimal gland secretion consists of various microbicidal proteins such as lysozyme, lacritin, immunoglobulins, and cytokines. The lacrimal gland also posses the IgA-secreting plasma cells, which protects the ocular surface from invasive pathogens [13].

#### 2.1.4. Meibomian Gland

Meibomian glands are sebaceous glands located in the eyelids. The specialized meibomian glands are composed of mebiocytes forming the acini that accomplish the process of lipogenesis and production of mebium. The meibum slowly seeps out of the eyelid margin during the gentle pumping process of blinking. The lipids secreted by the meibomian gland include cholesterol, cholesterol esters, wax esters, triglycerides, phospholipids, free cholesterol, and free fatty acids. The glandular secretion with the lipids and proteins mixture maintains the osmolarity, stability and prevents evaporation of the tear film [14,15].

#### 2.1.5. Eyelids

The eyelids contribute the first line of defense to the eye as they have mobile mucosal lining which entirely covers the ocular surface. Apart from the physical defense the eyelids are involved in the uniform distribution of the glandular secretion into the tear film protecting the ocular surface from desiccation [16].

### 2.2. Other Specific Components of the Ocular Surface Microenvironment

#### 2.2.1. Tear Film

The tear film can be considered as a special form of extracellular matrix component of the ocular surface. It is normally described to possess three layers: the outermost lipid layer, the middle aqueous tears, and the innermost mucous layer [13,17].

The lipids maintain the surface tension, viscosity, elasticity and systematic packing order which aid the tear film to maintain the ocular surface integrity and reduce evaporation of underlying aqueous in the open eye. The lipid layer has been hypothesized to interact with the mucin and enhance the formation of thin aqueous film which spreads uniformly over the ocular surface [18,19].

The aqueous phase of the tear film is loaded with not just water that lubricates the ocular surface, but contains numerous proteins including cytokines, immunoglobulins, and growth factors thus considered crucial in cell signaling and rehabilitation of the ocular surface during disease conditions. The aqueous phase also contains electrolytes and maintain the tear osmolarity [20].

Mucus, produced by the conjunctival goblet cells, is chiefly composed of mucins, immunoglobulins, salts, urea, enzymes, glucose and leukocytes [21,22]. The mucin layer is in contact with the epithelium and acts as surfactant by evenly spreading the tear film on the ocular surface. The soluble mucins interact with the transmembrane mucins to form the stable glycocalyx layer over the epithelium. The mucin layer harbors the commensals of the ocular surface and depletion of the mucin can alter the microbiome [20,23]. Mucins that have been detected in the ocular surface are MUC1, MUC2, MUC4, MUC5AC, MUC7, MUC13, MUC15, MUC16, and MUC17. Apart from integral backbone: water, protein and lipids the tear film is composed of electrolytes, vitamins and anti-microbial peptides [24,25]. Compromise in any layer of the tear film can potentially disrupt the ocular surface health.

#### 2.2.2. Immune Cells

The OSM is shielded by heterogeneous population of immune cells. The immune system is central to host protection, designed to respond efficiently to environmental and pathogenic insults. The ocular surface-associated lymphoid tissue (EALT) is composed of mucosal immune systems: conjunctiva-associated lymphoid tissue (CALT) and lacrimal drainage-associated lymphoid tissue. The ocular surface immune system is tightly regulated by the coordinated effort of the innate and adaptive immune responses. The innate immune system is the first-line of defense and functions to control initial infection and coordinate the adaptive immune response. Antigen-presenting cells (APCs) activated by a multitude of stimuli may present self-antigen to autoreactive T cells during the development of autoimmunity. APCs are classified as professional (DCs, macrophages, B cells, and some epithelial cell types) or on-professional (e.g., fibroblasts) in ocular surface. The adaptive immunity of the ocular surface depends on the cellular defense, mediated by the T cells, and the humoral defense mediated by the immunoglobulins secreted by the plasma cells [26,27]. The T lymphocytes are the residents of the ocular surface which consist of CD4+ and CD8+ cells also known as the interepithelial lymphocytes (IEL). They possess two unique receptors, the α/β T-cell receptor that aid in antigen recognition. The CALT is also composed natural killer (NK/NKT) cells a major subset of IEL population. T-helper (Th) cells comprises of diverse subpopulations including Th1, Th2, Th3, Th17 and Th memory cells based on the cytokine response triggered. Granulocytes are recruited from the blood circulation during adverse conditions. Environmental insults to the ocular surface sensitizes the corneal and conjunctival APCs resulting in the production of Th effector cells which release inflammatory cytokines amplifying the process of immune response and leading to ocular surface epithelial damage.

To maintain the immune homeostasis, the ocular surface tissues contain a variety of factors and different subsets of regulatory T cells to reduce inflammation-induced pathology in the lacrimal functional unit [28,29]. The anti-inflammatory cytokine in ocular surface includes TGF-β, IL-10, IL-RA, sIgA, etc. [28,29]. Immunosurveillance by CD4+ CD25+ Foxp3+ T cells, CD8+ T cells, γδ T cells, and NK/NKT cells present within the CALT of healthy subjects may provide protection against autoimmunity [28,29,30]. Though the immunopathogenesis of ocular surface autoimmune diseases remain largely unknown, a growing body of evidence suggest that a combination of excessive ocular environmental stress and/or immunoregulatory dysfunction, combined with genetically predisposed factors and/or hormone imbalance provides an environment conducive to activation of autoreactive lymphocytes. These autoreactive T and B cells are the basis of autoimmune-mediated pathology, such as DED, Mooren’s ulcerative keratitis, ocular cicatricial pemphigoid, etc. [28,29].

#### 2.2.3. Nerve Supply of the Ocular Surface

The ocular surface is populated with nerve fibers that are derived from the branches of the trigeminal nerve. They can maintain the action of blinking and tear reflex. Nerve endings secrete neurotransmitters and nerve growth factors (NGFs) which is essential in maintaining the epithelial integrity, proliferation and wound healing [31]. The lacrimal gland is supplied with the preganglionic parsympathetic nerve. The secretion of proteins, electrolytes and water by the lacrimal gland depends on neurotransmitters (acetylcholine and norepinephrine) released by the activation of either the parasympathetic or sympathetic nerves [13,32]. Apart from the parasympathetic nerves from the pterygopalatine ganglion, the mebomian gland is also supplied with the sympathetic nerves originated from the superior cervical ganglion and sensory fibers originated from the trigeminal ganglion [33]. Parasympathetic and sympathetic nerves as well as muscarinic and adrenergic receptors are present in conjunctival goblet cells, playing an important role in maintaining their physiological functions [34]. Thus, the innervations of the ocular surface contribute to the major components of the tear film.

#### 2.2.4. Systemic Hormones

The circulating hormones around the ocular surface are a pivotal factor involved in the maintenance of the ocular surface homeostasis [35,36]. Lacrimal and meibomian glands possess the sex hormone receptors. The androgens are capable of exerting a significant effect on the gene expression, protein synthesis, and immune response of the cornea, conjunctiva and the secretory functions of the lacrimal and meibomian gland [37,38,39]. Androgens exert an anti-inflammatory role with the synthesis of TGF-β associated with reduction of interleukin-1β and TNF-α, thus supporting lachrymal gland functions [40]. Growth hormone also plays an important role in regulatingthe meibomian gland size and morphology, cornea epithelium migration except for lacrimal gland [41,42,43,44]. Other hormones such as corticosteroids insulin, thyroid hormones and melatonin also play crucial role in the maintenance of the ocular surface [45,46]. Therefore, the systemic hormones can be considered as important component of the OSM.

#### 2.2.5. Vascular and Lymphatic Systems

The trafficking of immune cells, fluid balance, vitamin absorption and lipogenesis involves the role of lymphatic vasculature from peripheral tissue to draining lymph nodes [47,48]. The ocular surface vasculature is mainly observed in the conjunctival, episcleral layers and the limbal region [49]. The normal human cornea is avascular, yet it is nourished by the components of the blood. Arterial supply of conjunctiva originates from the peripheral tarsal arcades, marginal tarsal arcades and the anterior ciliary arteries. The blood and lymphatic vessel formation is primarily maintained by the vascular endothelial growth factors (VEGF) [50]. Similar to other organs, uninterrupted blood supply is required for the ocular surface to mediate the transport of growth factors, immune response and oxygen supply.

#### 2.2.6. Ocular Surface Microbiome

Microbiota was not considered crucial in maintaining the homeostasis of the ocular surface, until recently the metagenomic platform was successfully applied to identify various non-cultivable bacterial genome in the ocular surface. Dong et al. observed 24 genera including pathogenic and non-pathogenic bacteria in the ocular surface [51]. The microbiome of ocular surface also harbors viruses such as the herpes simplex type 1, hepatitis B virus, hepatitis C virus and Torque teno virus [52,53]. Similar to the commensal colonizing the gut and the skin, the ocular flora coexists with the components of ocular surface rendering immune tolerance and elimination of pathogenic microbes. Alteration in the microbiome status of the ocular surface can significantly affect the homeostasis through quorum-sensing mechanisms [54].

A common factor that modulates the microbiome is the exposure of ocular surface to antibiotics. Long term exposure to antibiotics can influence the reduction of the diversity and the burden of ocular surface commensals [55,56]. The ocular commensals participate in plethora ways to maintain the ocular hemostasis [57]. They shield the ocular surface from the pathogenic microbe; the bactericidal status of ocular surface is enhanced by priming the innate immune response, as the commensals are source of peptidoglycans; mucolytic enzyme producing commensals are involved in the mucin turnover rendering bacteriostatic activity of the tear film [58]. Thus, the alteration of microbiome ecology significantly affects the ocular homeostasis and the shift can also aid in the prognosis of future ocular surface related diseases.

### 2.3. Complexity and Integrity of the Ocular Surface Microenvironment

A healthy ocular surface microenvironment, especially a stable tear film, is essential to preserve the smooth optical surface, epithelial cell health, ocular comfort, provide protection from environmental and microbial insults. Interconnection between the ocular surface tissues and secretory glands through the central nervous and endocrine system directs production of the tear film, has evolved a complex network to maintain ocular surface microenvironment homeostasis, especially tear film stability [2,59]. As aforementioned, the ocular surface is a functional unit composed of the ocular surface tissues (cornea epithelium, limbus stem cells, conjunctiva, eyelids) and the tear-secreting machinery (the primary and accessory lacrimal glands, meibomian glands, conjunctival goblet cells and epithelial cells) [2,59]. These elements work together through nervous communication and systemic hormones to maintain microenvironment homeostasis of the ocular surface [2,59].

The lacrimal functional unit is tightly controlled by neural input from the ocular surface tissues, especially the cornea [60]. Subconscious stimulation of the free nerve endings rich in the cornea, triggers afferent impulses through the ophthalmic branch of the trigeminal nerve (V), which then integrate to the central nervous system and the paraspinal sympathetic tract to stimulate tear production. The efferent branch of the loop extends fibers via the pterygopalatine ganglion and then to the primary and accessory lacrimal glands, meibomian glands and conjunctival goblet cells [2,59]. Thus, the efferent branch of the loop can stimulate the three major tear film components (lipid, aqueous and mucin) secreted in coordination, on the ocular surface to maintain ocular surface microenvironment homeostasis.

Based on our current understanding about the ocular surface microenvironment, we can develop a unified theory about the integrity and function of the lacrimal functional unit. The fundamental function of homeostasis of ocular surface microenvironment is to maintain the tear film stability. The lacrimal functional unit acts as an integrated servomechanism. If one or more components of the lacrimal functional unit are compromised, the entire functional unit can enter the dysfunctional state [2,59].

Tear secretory function can be disrupted by disease of the afferent, efferent, or glandular components of the lacrimal functional unit, as well as from ocular surface or glandular inflammation [2,59]. Dysfunction of components of the lacrimal functional unit results in tear film compositional change, with decreases in components that promote tear film stability, increases in tear film osmolarity, consequent inflammation and evaded levels of derivative components [2,59]. Osmotic stress to the ocular surface is sufficient to activate MAPK and nuclear factor (NF)-κB, and to stimulate the production of variety of inflammatory mediators including interleukin (IL)-1β, tumor necrosis factor (TNF)-α, IL-8 and a number of matrix metalloproteinases (MMPs) by the ocular surface epithelium [28,29]. Epithelial-derived inflammatory cytokines can activate immature resident dendritic cells, and trigger the initial events leading to localized autoimmunity [28,29]. Irrespective to the initiating point of etiology, once the compromise of the ocular surface microenvironment develops, inflammation becomes the key mechanism contributing to the cause and consequence of ocular surface damage [28,29].

The release of inflammatory cytokines from lymphocytes and ocular resident cells can obstruct the tearing reflex by interfering with neurotransmitter release and the response of the tear-secreting machinery to the neurotransmitters [2,59]. IL-1β has been shown to cause the release of opioids that bind to opioid receptors on neural membranes and inhibit neural function in ocular surface and lacrimal gland [61]. IL-2 has also been shown to bind directly to the delta opioid receptor in peripheral nerve cells inhibiting cAMP production and thus neural function [62].

In addition to regulating the function of lacrimal glands and Meibomian glands, androgens have been shown to play a pivotal role in suppressing T lymphocytes and supporting the function of the regulatory T lymphocytes by up-regulating TGF-β [63]. Thus, sex-steroid imbalance, especially reduction in androgen levels, may predispose individuals to the development of dry eye and autoimmune condition affecting the ocular surface [36].

## 3. Ocular Surface Microenvironment Change in Dry Eye

A single component change can eventually break down the homeostasis of the ocular surface microenvironment, by initializing the pathological change of DED, and can promote the vicious cycle of the disease. Therefore, it is important to distinguish the driving force of the pathological changes in dry eye. To achieve this, complete analysis of history and careful examination of the OSM should be conducted on the patients. In this part, we focus on the tissue, cell and matrix change in dry eye and how these changes are related to the pathophysiology of DED.

### 3.1. The Change of Tissue Components of Ocular Surface Microenviroment in Dry Eye

#### 3.1.1. Cornea

Corneal scarring, opacification, and degeneration are the noted complications of the dry eye. Desiccation stress and amplified inflammatory response in dry eye significantly alters the thickness of the central and peripheral cornea contributing to corneal thinning [64]. The imbalance between the matrix metalloproteinases (MMPs) and the inhibitors of MMPs significantly degrade the extracellular matrix of the collagenous stroma resulting in corneal thinning [65,66]. Deterioration of the tear film can result in irregular corneal surface that affects the visual quality. Higher order aberrations are focused in the anterior cornea than in the posterior corneal surface when the tear interface is compromised [67]. Dry eye is also characterized by reduction of superficial corneal epithelial microvilli, and the conversion of corneal epithelial phenotype to Keratin 10 positive epidermal phenotype which results in tear film instability [68,69]. Neovascularization, pannus formation, and ulcers are frequently observed in the late stage of severe dry eye, which may lead to blindness. The density of corneal endothelium cells is significantly reduced in central cornea in mild to severe dry eye [70].

#### 3.1.2. Conjunctiva

Chronic inflammation of the conjunctiva is commonly present from mild to severe DED. A major pathological change of conjunctiva in response to deficiency of tear film and chronic inflammatory response is squamous metaplasia. Goblet cell density also dramatically decreases in DED [71,72]. Conjunctivochalasis, described as lax and redundant folds of bulbar conjunctiva, has been shown associated with the symptoms and clinical signs of DED by decreased tear stability, pooling of tears [73]. Pterygium can cause reduced tear breakup time thus inducing DED [74,75].

#### 3.1.3. Lacrimal Gland

Dysfunction of lacrimal gland can be induced by various reasons such as aging, prolonged exposure to visual display, dry environments, radiation therapy, contact lenses, refractive surgery, hormonal imbalance, ocular cicatricial pemphigoid, Sjögren’s syndrome and systemic disorders [76]. Age related consequence of the lacrimal gland functions is the progressive acinar atrophy, uncontrolled fibrosis and infiltration of the lymphocytic. Autoimmune disorders such as Sjögren’s syndrome substantially target the lacrimal gland, wherein autoantigens are expressed on the surface of the epithelial cells, leading to the retention of CD4 and CD8. The lymphocyte aggravates the immune response resulting in loss of lacrimal acinar and ductal cells [77]. The autoantibodies also targets and binds to the M3 acetylcholine receptors expressed on the secretory acinar cells inhibiting the neural stimulation [78]. Diminished glandular secretion in DED could be due to the impotency of the lacrimal acinar cells and the ductal cells to respond to the neuroendocrine stimuli [79].

Obstruction of the lacrimal gland is often observed in patients with graft-versus-host disease, wherein the acinar and the ductal lumen is obstructed by the accumulation of granules, fibrosis and cellular debris leading to tear deficiency [80]. Radiation therapy used in cancer treatments can lead to transient or permanent dysfunction of the lacrimal gland due to the apoptosis of the lacrimal acinar cells [77]. Thus, the primary irreversible changes in the lacrimal gland, i.e., loss of secretory acinar cells, fibrosis and gland atrophy affects the quantity and quality of the glandular secretion resulting in severe aqueous deficient DED.

#### 3.1.4. Meibomian Gland

The lipids present in the mebium have an important role in the maintenance of the ocular surface and fluctuation in the quality and quantity of lipids reflects changes in the tear components thereby proceeding to evaporative DED. Lipid order and packing in the mebium during MGD is altered. Over expression of protein and under expression of the lipid moieties reflect on the viscosity of the mebium expressed leading to obstruction of the gland. Triglycerides are proved to be elevated in the tear of dry eye patients and are the targets of reactive oxygen species contributing to the conformational changes in the lipid moieties. The lipid peroxidation markers, 4-hydroxy-2-nonenal (HNE) and malondialdehyde (MDA) are elevated in case of MGD [81].

MGD is classified into hypersecretory MGD, hyposecretory MGD and obstructive MGD among which obstructive MGD is considered to be the commonest form. The obstructive form of MGD may be congenital or may be influence by factors such as age, sex, hormonal imbalance, systemic diseases, autoimmune disease and topical medication. Progressive obstruction of the ducts due to intraglandular cystic dilatation, hyper-keratinization widens the duct leading to gland drop out, tear film instability excessive evaporation, tear film stability, hyperosmolarity, and desiccating stress [82,83]. MGD alters the levels, ratio, conformational changes of individual polar and non-polar lipid moieties, which affects the lipid–lipid interaction and the lipid–protein interaction eventually progressing to evaporative DED.

#### 3.1.5. Eyelids

Reduction in the blinking frequency decreases the thickness of the lipid layer leading to increased evaporation of the aqueous layer [84]. Age-related degenerative changes, trauma, facial palsies, lagophthalmus, proptosis or the floppy eyelid syndrome can influence the eyelid laxity resulting in inefficient and infrequent blinking. Thus, the altered eyelid laxity is incapable of exerting muscular pressure over the meibomian glands to release the meibum, which results in MGD and DED. Inefficient blinking also proceed in the accumulation of toxin, pathogen and foreign body as tear clearance solely depends on the frequency of blinking at uniform intervals. Incomplete blinking affects the uniform distribution of tear on the ocular surface, which results in shortening of the tear film break up time. DED in turn will result in keratinization, thickening, neovascularization, and inflammation of the eyelid [85,86]. Blepharitis is the inflammation of the eye lid associated with bacterial infections caused by *Staphylococcus aureus*, *S. epidermidis* and *Corynebacterium* spp. Bacterial biofilm and the excreted virulence factors aggravate blepharitis leading to hyperemic, edematous, ulcerated eyelids, recurring chalazia resulting in the obstruction of the meibum which contributes to DED [87].

### 3.2. The Changes of Other Specific Components of the Ocular Surface Microenvironment in Dry Eye

#### 3.2.1. Tear Film

Tear hyperosmolarity, tear film instability, excessive tear film evaporation, delayed tear clearance, infrequent and ineffective blinking are major contributing factors to the etiology of DED. Hyperosmolarity is a condition featured by dehydration resulting in increased osmotic concentration and is characteristic of DED [88]. Hyperosmolarity can usher the apoptosis of the corneal epithelial cells [89], and induce pro-inflammatory stress to the human limbal epithelial cells [90]. A comprehensive summary of causes and effects of tear film hyperosmolarity in dry eye is provided in the 2007 DEWS [1].

Tear film instability may be the consequence secondary to hyperosmolarity or may be the prime event. The factors influencing the stability of the tear film includes reduced tear production, delayed tear clearance, reduced quantity and quality of the lipid layer, compromised tear components, ocular surface irregularities and ocular surface inflammation. Tear film instability is the sequel of a series of events, which initiates from the lipid layer of the tear film. Deficient lipid layer induces evaporation, insufficient lipid binding proteins in the aqueous phase cripples lipid transport eventually the glycocalyx layer loses its hydrophilic property due to dehydration resulting in ruptured tear film. Areas of ocular surface may remain non-wettable due to the disproportional layer of ruptured tear film eventuating squamous metaplasia [91].

Excessive tear film evaporation is considered to be an important cause of tear hyperosmolarity, causing ocular surface inflammation [1]. Previous studies pointed out that tear film evaporation rates increased in dry eye patients [92], typically in association with a loss of integrity of the tear lipid layer [93]. Meibomian gland dysfunction and anterior blepharitis are the leading cause of the lipid layer integrity disruption [94]. Environmental conditions, tear film instability, age, sex and wearing contact lens have been reported to affect tear film evaporation rate [95,96,97,98].

Delayed tear clearance increases the toxic cell waste products, environmental antigens and inflammatory mediator concentrations such as interleukin (IL)-1, tumor necrosis factor-α (TNF-α) and MMP-9 in the tear film and contribute to the pathogenesis or aggravate the severity of dry eye [99,100]. Reduced tear clearance would cause ocular surface epithelial changes [101].

Every layer of the tear film has an important role in the homeostasis of OSM, fluctuation in the quality and quantity of the tear components thereby proceeding to DED. Table 1 summarizes the tear film components compromised in DED.

#### 3.2.2. Immune Cells

In DED, the OSM experiences failure of immunohomeostasis, resulting in chronic inflammation. Inflammatory response in DED is an increscent process which involves: (1) activation of mitogen-activated protein kinase (MAPK) and nuclear factor (NF)-κB in the ocular surface epithelium by osmotic stress; (2) release of pro-inflammatory cytokines and chemokines including IL-1β, TNF-α, and IL-8 and a number of MMPS (MMP-1, -3, -9, -10, and -13); (3) up-regulation of MHC class II on the ocular surface epithelial surface; (4) mobilization and activation of ocular surface APCs; (5) recruitment of Th1 and Th17 cells to the inflammatory site; and (6) amplification of immune response and forming of a vicious circle [28,29].

CD4+ T cells play a primary role in the immunopathogenesis of DED. As noted, activated CD4+ T cells are localized within the ocular surface tissues of dry eye patients. Desiccating stress-activated CD4+ T cells, when adoptively transferred to T-cell nude mice, were sufficient to elicit autoimmune DED [30,110,111]. Th1 cell-produced IFN-γ plays a pivotal role in desiccating stress—induced epithelial apoptosis, goblet cell loss, and squamous metaplasia in ocular surface [72,110,112]. Th17 cells are involved in the secretion of cytokines such as IL-17A, IL-17F, and IL-22 which are associated with increased MMP-3 and MMP-9 levels and are involved in the corneal barrier dysfunction in DED [72,113]. CD4+ CD25+ Treg cells are involved in the suppression of the autoreactive T cells by producing anti-inflammatory cytokines, IL-10 and TGF-ß and suppressing the organ specific autoimmunity. CD8+ Treg among the subsets of Treg cells play an immunosuppressive role targeting the corneal epithelial cell disruption and autoimmunity [113]. The NK cells are capable of inducing damage directly to the ocular surface or by activating the APC through the release of IFN-γ and promoting the production of IL-17A in DED [114]. Dysfunction of regulatory T cells in suppressing CD4+ T generation is noted in DED [30,111,114].

#### 3.3.3. Nerve Supply of Ocular Surface

The DED corneas are characterized by reduced subrabasal density and altered morphology (nerve sprouts, tortuosity). This is due to the epitheliopathy of the ocular surface in DED unshelters the ocular nerves to inflammatory cytokines stimulating the neurotrophic growth factors resulting in neuropathies [115,116]. The parasympathetic nerve endings release acetylcholine the major neurotransmitter into the synaptic cleft acetylcholine binds to the muscarinic cholinergic receptors (M3AChR) present on the effector cells to transmit the signals. Excessive levels of cytokines in DED inhibit the release of acetylcholine from the nerve endings and auto autoantibody blockade of M3AChR, disturbing the neuronal stimuli transmission to the secretory component of the OSM [117,118]. Moreover, diabetes can cause lachrymal and corneal neuropathy, and ultimately induce the change of OSM and dry eye [119,120]. Some ophthalmologic surgeries such as refractive surgery and cataract surgery could cause corneal nerve transection which results in decreased feedback to the lacrimal gland leading to reduced tear production [121,122,123].

#### 3.3.4. Systemic Hormones

Hormones play an important role in ocular surface homeostasis. Androgen deficiency could lead to obstructive MGD with a lack of lipids at the lid margin and in the tear film, an altered lipid profile, and dry eye symptoms [124,125]. While estrogen deficiency is related to post-menopausal DED by initiating tissue-specific apoptosis [126]. Thyroid hormones state disorders could also induce ocular surface inflammation, dry eye symptom and MGD [127].

#### 3.3.5. Vascular System

During immune response the afferent lymphatic vessels and efferent blood vessels occur in concert to protect the ocular surface. Angiogenesis is the formation of new blood vessels, which involves either lymphangiogenesis or hemangiogenesis, where in the DED, is characterized by lymphangiogenesis. Chronic inflammation in DED triggers the macrophages of the ocular surface, which orchestras lymphangiogenesis by two different mechanisms: (1) the CD11b^+^ macrophages infiltrate the cornea and transdifferentiate into the endothelial lymphatics enhancing lymphangiogenesis; and (2) macrophage activates the NF-κB signaling and promotes the expression of vascular endothelial growth factor (VEGF) C and VEGF-D and associated receptors VEGFR-2 and VEGFR-3 contributing to the process of lymphangiogenesis [128]. Lymphangiogenesis of lacrimal gland was observed in the experimental models of DED by the activation of Dll4/Notch signaling pathway and hypoxia-inducible factor-1 α (HIF-1α) [129]. Moderate to severe DED induces neovascularization of the cornea and corneal pannus can develop in the late stage of Stevens-Johnson syndrome. Studies on murine DED model has shown that low-grade inflammation associated with DED can potentially induce lymphangiogenesis in cornea without accompanying hemangiogenesis [128].

#### 3.3.6. Ocular Surface Microbiome

Compromise in the ocular microbiome is evident in conditions such as DED, contact lens wear, keratoprosthesis, antibiotic exposure and infectious states. The ocular surface integrity is breeched in DED, promoting the flora to exert its pathogenic effect and activating triggering the innate immune response. Prevalence of *Staphylococcus aureus* and coagulase negative *Staphylococcus*, *Corynebacterium* and *Propionibacterium* was observed to be associated with DED [57,130]. The alteration in ocular microbiome in patients with SJS exposes their ocular surface to infection and the risk of predisposition increases during ocular surgeries. The rate of culture positivity rate of microorganism was observed to be higher in eyes affected by DED, indicating their opportunistic contribution towards the pathogenesis. In addition to coagulase negative *Staphylococcus aureus*, *Escherichia coli*, and *Streptococcus pneumonia* isolated from eye in SJS exhibited resistance towards second-generation flouroquinolones [131]. Altered ocular microbiome influence the ocular autoimmunity, impairs innate immunity functions and decrease the IgA production which promotes the entry of pathogens to the ocular surface [132]. Studies on DED ocular microbiome are very sparse and poorly understood. The emerging field of ocular microbiome can promote awareness on the antibiotic policies prescribed post-surgery in DED patients.

## 4. Dry Eye Management: Ocular Surface Microenvironment Targeted Therapy

The DEWS committee has enumerated DED treatment recommendations based on the severity levels, and the evaluation of treatment outcomes mainly depends on the subsiding symptoms [133]. However, recent studies reported that there is no consistent relationship between common clinical signs and symptoms in dry eye disease [134]. Among patients with MGD, the most prevalent form of evaporative dry eye, asymptomatic MGD is more common than symptomatic MGD, therefore symptom-based approaches in the diagnosis and management of dry eye may be inappropriate [135].

Based on our understanding on OSM, we recognized that changes of different components rather than LFU could cause compromise of OSM and result in DED. On the other hand, dry eye will aggravate the pathological change of the OSM components. Therefore, in-depth investigation on the OSM components will help us to unravel the etiology and pathophysiology of dry eye in specific patient. Here, we propose that dry eye treatment strategy may need to target on the specific OSM components which are compromised in dry eye. Table 2 summarizes the ocular surface microenvironment targeted therapy for dry eye.

### 4.1. Therapy Targeting Cornea

DED tear film is characterized by down regulation of essential growth factors such as TGF-β, EGF, PDGF, KGF, and HGF, which are involved in the proliferation and migration of epithelial cells and in the process of wound healing. Autologous serum eye drops are loaded with EGF, HGF, vitamin A and fibronectin mimicking the tear film which lubricate and maintain the integrity of the ocular surface. Thus, autologous serum can induce proliferation, migration, restore the tight junctions of the ocular surface epithelial cells aiding in wound healing. Application of autologous serum in DED showed prominent improvement in the tear film stability and corneal wound healing [136,137]. Topical application of EGF could improve the stability of tear film and the integrity of epithelium [138].

Human amniotic membrane has greatly benefited the ocular surface by releasing bioactive factors. Topical application of amniotic membrane extract could help in stabilizing the tear film by maintaining the integrity of epithelium and alleviate the ocular surface inflammation in dry eye [139]. Amniotic membrane implanted as a therapeutic contact lens is an effective and safe method to treat epithelial defects [140]. In severe dry eye, application of contact lenses may help to decrease corneal epitheliopathy, improve visual acuity, comfort, and assist to prevent progressive corneal epithelial defect [141,142]. The contact lenses also act as a carrier for sustained release of the drugs in ocular diseases [143]. Various in vitro and animal model studies have attempted to synthesize inhibitors of MMP-9 in treating DED. However, there is no specific approved MMP-9 inhibitor for DED treatment. Although not a targeted therapy, the anti-inflammatory therapy drugs such as doxycycline, azithromycin, cyclosporine A and corticosteroid dexamethasone are proved to inhibit the gelatinolytic activity of MMP-9 [144,145,146]. Similarly, osmoprotectants l-carnitine, erythritol and betaine are proved to inhibit the expression and activation of MMPs [147].

### 4.2. Therapy Targeting Conjunctiva

The squamous metaplasia of conjunctiva epithelium can be treated similar to the cornea as corneal described above. Vitamin A deficiency in the ocular surface leads to hyperkeratinization of epithelium, squamous metaplasia and decreased conjunctival goblet cells [148]. Topical vitamin A is found to be a promising approach in treating DED, as it improves the tear film stability by enhancing mucin production, thus maintaining the functionality of ocular surface epithelial cells [149]. P2Y2 receptor purinergic receptors located in the conjunctival goblet cells, acinar, ductal epithelial cells of the meibomian gland. P2Y2 receptors are activated by ATP and UTP and are studied to be involved in cell proliferation, apoptosis and inflammation [150]. Diquafosol tetrasodium is a P2Y2 agonist which was found to increase the tear film stability, hydrate the ocular surface by inducing tear fluid secretion from the lacrimal glands and mucin secretion by the conjunctival goblet cells [151]. Hydroxyeicosatetraenoic acid (15(S)-HETE), rebamipide and Gefarnate are secretagogue that can induce the secretion of mucin layer by the conjunctival goblet cells [152]. Treatment on pterygium and conjunctivochalasis could improve the dry eye symptom in these patients.

### 4.3. Therapy Targeting Lacrimal Gland

Primary Sjögren’s syndrome is often accompanied by deteriorated secretory glands mainly the lacrimal gland, hence the therapy targets to improve the glandular secretion and to prevent dryness. B-lymphocytes activated by the B cell activating factor (BAFF) secrete autoantibodies/cytokines and involve in the pathogenesis of the disease. Thus, the various monoclonal antibodies are designed to suppress the B-activation. Efficacy of various immunomodulators such as rituximab, abatacept, belimumab, infliximab, etanercept and hydroxychloroquine have been studied, yet the outcome varies in each study and these are under development [153,154,155]. The current treatment strategy is symptom based recommending the use of artificial tear, topical cyclosporins, secretagogue [156]. Neurostimulation has been recently studied on animal models which directly target the lacrimal nerve enhancing the aqueous tear secretion. The novel strategy was found to be effective in increasing the aqueous tear volumes [157]. Next generation regenerative medicine has advanced in their focus towards the transplantation of bioengineered lacrimal gland. Hirayama et al. using the organ germ method has established and engrafted bioengineered lacrimal gland in a murine model, which showed significant efficacy in restoring the secretory function and enhancing the tear production [158].

### 4.4. Therapy Targeting Meibomian Gland

Warm compresses, manual lid massage, and gland expression can be prescribed to patients with meibomian gland dysfunction. Various mechanical devices have been developed to express meibum, among them is FDA approved Lipiflow System™ which liquefies meibum by thermal compression [159]. Intense-Pulsed-Light (IPL) consist of a light source emitting high-intensity polychromatic light of wavelength ranging from (515–1200 nm). The high intensity light is focused on to the target tissue resulting in the release of heat aiding in lesion removal. Using the similar principal periocular IPL devices are currently available for treating MGD related DED [160]. Lipid containing lubricants are also used in treating DED to compensate the lipid layer of the tear film. The active ingredients in these lubricants include light mineral oil, mineral oil, castor oil, glycerin, and polupropylene glycol at varying concentrations. Phospholipid liposomal sprays used on the lid margin can consort with the meibum to maintain the polar lipid layer and improve the spreading of the lipid layer. Oral dietary supplementation of ω-3 fatty acid is emphasized in MGD patients as the dietary lipid intake can alter the meibum composition. Clinical studies have observed the improvement of tear stability, and tear secretion in patients with MGD after the use of ω-3 fatty acid [161]. Combinational therapy of topical lipid emulsion, eye lid cleansing wipes and oral ω-3 fatty acid has also shown significant improvement in the functionality of the meibomian gland in MGD patients [162].

### 4.5. Therapy Targeting Eyelids

Eyelid hygiene, antibiotics and warm compress are the traditional therapy recommended according to the severity of blepharitis. Oral or topical azithromycin is also prescribed as an adjuvant for the patients with blepharitis [163,164]. When the severity of the syndrome increases and the topical treatment interventions fail, minor eyelid surgical options are suggested. For example, temporary tarsorrhaphy, reduces the opening of the eye and prevents the tear evaporation [165].

### 4.6. Therapy Targeting the Tear Film

The initial step in the management of dry eye is focused to restore the volume of the tear and compensating the tear components. The down regulated tear film components are rendered to the ocular surface exogenously by various forms of eye drops or ointments in the management of DED.

#### 4.6.1. Compensation of Tear

Artificial tears are the most common initial therapeutic strategy for DED, and are mainly composed of ingredients that mimic the tear to maintain the osmolarity of the depleted tear film. Preservatives such as benzalkonium chloride should be avoided if possible, because they induce toxic epithelial damage and accentuate inflammation [166]. Electrolytes such as potassium and bicarbonate ions are supplemented to maintain the ionic balance of the tear film. Bicarbonates and potassium ions enable the rehabilitation of corneal epithelial barrier and the deficient mucin layer [167]. Hypotonic artificial tears with glycerin, erythritol, and levocarnitine aid in the maintenance of tear osmolarity in DED. Water soluble polymers such as hypromellose, hydroxyethylcellulose, methylcellulose, carboxymethylcellulose, hyaluronic acid, polyethylene glycol, propylene glycol, glycerine, polysorbate and polyacrylic acid act as lubricant in the reduction of friction and irritational discomfort. The polymer also enhances the viscosity and increases the retention time and mucoadhesion of the artificial tears on the ocular surface. The hydroxyl or carboxyl functional groups of the polymers interact by forming hydrogen bonds with the water molecules on the ocular surface [168,169]. Lipid containing lubricants are also used in treating DED to compensate the lipid layer of the tear film. The active ingredients in these lubricants includes light mineral oil, mineral oil, castor oil, glycerin, polupropylene glycol at varying concentrations [170]. Phospholipid liposomal sprays used on the lid margin can consort with the meibum to maintain the polar lipid layer and improve the spreading of the lipid layer [171]. An alternative approach to synthetic artificial tears is autologous serum. For some severe DED patients especially the treatment of artificial tears could not work, diluted autologous serum can mimic natural tears more closely [172].

#### 4.6.2. Stimulated Tear Production

Stimulation of the secretory gland and the secretory cells are upcoming treatment modalities in DED. The therapy involves the stimulation of lacrimal gland by inducing the cholinergic signaling pathway in the acinar cells. Pilocarpine (parasympathomimetic alkaloid) and cevimeline are the muscarinic agent acting as an agonist on M1 and M3 receptors increasing the tear dynamics, but are associated with sweating and diarrhea, and may additionally cause accommodative spasm and brow ache in young patients [168,173]. Diquafosol tetrasodium, a P2Y2 agonist, that stimulate transconjunctival water flow and mucin secretion from conjunctival goblet cells, exhibits therapeutic efficacies and good tolerability in patients with DED [174], but also accompanied by side effects such as sweating, headache, diarrhea, nausea, abdominal pain [151]. Pituitary adenylate cyclase-activating polypeptide (PACAP) is a neurotransmitter through the PAC1-R/cAMP/PKA/AQP5 signaling pathway can stimulate the lacrimal gland and induce tear secretion. Studies on the effect of topical PACAP on murine models have proved that it is capable of regulating the tear protein secretion as well as tear fluid secretion [175]. Its clinical safety, ocular tolerability and therapeutic efficacies in dry eye patients need to be further confirmed.

The principle of neurostimulation therapy involves the activation of peripheral nerve pathway of the target organ to restore the organ functionality. Clinical studies have used the Oculeve intranasal neurostimulation device to activate the nasal sensory neurons using electrical stimuli in enhancing the tear secretion. The results showed that the neuro stimulator device was efficient to induce tear secretion and increased the tear volumes [176].

#### 4.6.3. Controlling Tear Evaporation

Lipid based artificial tears can protect the tear film evaporation, additionally modulating the atmospheric humidity and decreasing the ocular surface exposure can enhance the protective effect. The periocular humidity can be increased by using moisture-retaining eyeglasses, swimming goggles [177]. Apart from goggles, gas-permeable scleral lenses also retain the humidified environment and promote wound healing of the epithelial cells [178]. Prosthetic Replacement of the Ocular Surface Ecosystems (PROSE) are custom-designed lens made of fluorosilicone/acrylate polymers which are studied to be safe and effective towards various ocular surface disorders [179,180]. Though the sclera contact lenses are promising mode of DED therapy, the risk of microbial keratitis, difficulty in maintenance, handling becomes the retracting disadvantages. Additionally, castor oil eye drops are effective and safe in the treatment of MGD resulting from prevention of tear evaporation [181].

#### 4.6.4. Regulating Excessive Nasolacrimal Drainage

Punctal occlusions retain the exposure of lubricants and inhibit the drainage of natural tears. Punctal plugs are effective in patients with severe aqueous-deficient dry eye disease [182]. Punctal plugs are studied to reduce the hyperosmolarity, improve the goblet cell density and enhance the tear film stability [183]. However, the punctual plugs are piggy backed with certain drawbacks: plug extrusion may enlarge the puncta, punctal scarring, canalicular stenosis, displacement of plugs into the canalicular pathway leads to canaliculitis and dacryocystitis, pyogenic granuloma due to mucosal damage caused by plugs, epiphora, microbial contamination induce biofilm formation with chronic infection and may cause localized discomfort characterized with allergic reactions [152,184]. Blocking the puncta can also exposes the ocular surface to tear film loaded with pro-inflammatory cytokines which may intensify inflammation resulting in persistent ocular surface damage [185]. Thus, the contraindications should be taken into consideration while choosing punctual plugs in treating DED.

### 4.7. Therapy Targeting Inflammation

Regardless of the initiating etiology of DED, a common pathogenic mechanism of DED is related to a vicious circle of inflammation with consequent ocular surface damage. Anti-inflammatory drugs have gained importance as they disrupt the vicious cycle and restore the normal ocular homeostasis in DED. Different anti-inflammatory drugs have been applied in clinic to treat dry eye related inflammation.

Corticosteroids can suppress the expression of pro-inflammatory genes and enhances the expression of anti-inflammatory genes annexin-1 (lipocortin-1), IL-10 and the inhibitors of NF-*κ*B [186]. Topical corticosteroid use is highly effective on suppressing ocular surface inflammation in DED; however, due to a wide range of potential side effects including glaucoma, cataracts, and ocular infection, it is limited to short-term use (2–4 weeks) [187,188,189,190].

Nonsteroidal anti-inflammatory drugs (NSAIDs) have been evaluated as prospective therapy for dry eye. The anti-inflammatory properties of NSAIDs lie in blocking COX-1 and/or -2 which play an important role in the synthesis of prostaglandins from arachidonic acid. The topical formulations of NSAIDs including pranoprofen, bromfenac, and thymosin β4, have been confirmed to be effective on suppressing ocular surface inflammation and eye pain in DED [191,192,193].

Doxycycline and azithromycin are antibiotics that are considered to have anti-inflammatory property. Doxycycline can inhibit the activation of the MAPK and NFkB pathways and protects the corneal epithelial barrier function and conjunctival goblet cells [144]. Azithromycin can inhibit the inflammatory cytokines, chemokines and MMP-1, -3 and -9 produced in response to the desiccating stress [194].

Cyclosporine A (CsA) inhibits the T-cell activation thereby acting as an immunosuppressant. It can inhibit lymphocytic infiltration, decrease the immune, inflammatory response and inhibit apoptosis of the lacrimal and conjunctival epithelial cells in DED [195,196]. The commercial dose of CsA eye drops available is 0.05%, which is effective only in cases of mild to moderate dry eye. Tacrolimus (FK506) is another immunosuppressant capable of inhibiting the activity of calcineurin to dephosphorylate the nuclear factor of activated T cells [197]. Topical 0.03% tacrolimus eye drop can improve tear stability and ocular surface status in cases of Sjögren’s syndrome related DED [198].

Lifitegrast is a novel small molecule integrin antagonist that blocks the binding of intercellular adhesion molecule 1 (ICAM-1) to lymphocyte function-associated antigen 1 (LFA-1). Lifitegrast ophthalmic solution 5% was approved in the USA in 2016 for the treatment of DED. Phase III clinical trials demonstrated that lifitegrast was engineered to break the interaction between LFA-1 and ICAM-1, restricts T-cells activity and improving both signs and symptoms without obvious side effects in DED [199]. Based on the available evidence, inhibition of LFA-1/ICAM-1 interaction with lifitegrast offers a novel and effective approach to reducing ocular surface inflammation in DED [199,200,201].

Tofacitinib (CP-690,550) is a target specific inhibitor of Janus kinase (JAK) signaling pathway which is involved in the pathogenesis of inflammatory diseases. A phase I/II study demonstrated that topical application of tofacitinib improve both symptoms and signs in DED, and showed potential immunomodulatory effect by reducing the HLA-DR on the conjunctival cell surface and dampening the levels of inflammatory cytokines in tear [202,203].

Dietary supplements of essential fatty acids, such as fish oils, which contain ω-3 and -6 fatty acids, have been recommended for DED treatment. Fatty acids are eicosanoid precursors, known to reduce inflammation with decreased T-cell proliferation and inflammatory cytokine production [204]. It has been shown that fatty acids is beneficial in reducing symptoms and signs of ocular surface inflammation in DED [205].

### 4.8. Therapy Targeting Nerve Supply of the Ocular Surface

An important mechanism of DED is an altered ocular surface sensitivity leading to a neurosecretory block [3]. Nerve growth factor (NGF) and its receptor p75 and TrkA are highly expressed in the ocular surface and may play an important role in ocular surface diseases [206]. NGF can increase ocular surface sensitivity, inhibit inflammatory reactions, reduce the apoptosis of corneal epithelium and regulate tear film production by lacrimal gland and goblet cells, thus NGF can be considered a novel treatment for dry eye [207,208].

### 4.9. Therapy Targeting Systemic Hormones

Numerous studies state that the secretory function of the lacrimal and meibomian gland is regulated by the sex hormones. Hormonal supplementation in DED is also being widely studied and showed their role in improving the levels of secretion and suppression of inflammation [209]. Detection of androgen receptors expression on the ocular surface tissues has initiated the idea of synthesizing androgen and estrogen receptor inhibitors [210]. Growth hormone supplementation may be a novel therapy to treat corneal epithelial defect due to severe dry eye [42].

### 4.10. Therapy Targeting Vascular and Lymphatic System

Inflammatory response stimulates the lymphangiogenic factors VEGF-C and VEGF-D through pro-inflammatory cytokines (IL-1β and IL-17) signals. Studies using murine models showed that blocking of pro-lymphangiogenic VEGF-C by anti lymphangiogenic agents could potentially suppress inflammation and epitheliopathies associated with DED [211]. Doxycycline has also being demonstrated to be a potential therapeutic agent inhibiting VEGFC and suppress lymphangiogenesis in murine models which can be extrapolated to DED therapy [212]. For the conjunctivochalasis caused lymphangiectasia, subconjunctival bevacizumab injection and liquid nitrogen cryotherapy may be an effect therapy [213,214].

## 5. Conclusions and Prospective of Future Research

Dry eye is a multifactorial disease with complex pathophysiological process. The mechanism by which the OSM changes can result in the presentation of differential clinical symptom and signs is not clearly known til date. The interactions between different microenvironment components during the development of DED also need to be further illustrated. Our understanding on DED will undoubtedly increase further studies on OSM.

The ultimate goal of DED management is to restore the ocular surface and tear film to their normal homeostatic state, i.e., the healthy OSM. A wide range of therapeutics is available in treating DED, but we need to pay attention to evaluate whether certain treatment interventions can interfere with the functions of another OSM component, inducing unrelated complications or even exacerbating DED. For instance, puncta occlusion can alter the dynamic of tear film, and long-term application of artificial tear with preservatives can induce epithelial damage and alteration of the ocular surface microbiome. Customized and personalized therapy targeting the OSM will aid in complete resolution of DED.

## Figures and Tables

**Table 1:** Tear film components compromised in DED.

Layers of the Tear Film	Compromised Components Related to Tear Film Instability and Ocular Surface Homeostasis	
Lipid layer [81,102,103]	↓ Cholesteryl esters, ↓ Free fatty acids	Involved in evaporation retardation and possess surface-active properties
	↓ Wax esters	Bridge the polar and non-polar lipid phase, exert a condensing effect, due to the presence of the saturated fatty acid component
	↓ Ttriacyl glycerides	Act as a buffer system for the phospholipids to resist the surface pressure changes in the air water interface
	↓ Pphospholipids, ↓ Sphingomyelin, ↓ Cerebrosides	Maintains the integrity and arrangement of the non-polar lipids
Aqueous layer [104,105,106]	↓ Lipocalin	Involved in preventing corneal desiccation by scavenging lipids, removes fatty acid and phospholipid from the ocular surface
	↓ Lyzozymes	Act as an antibacterial agent by hydrolysing the β-(1-4) glycosidic linkages between bacterial cell wall carbohydrates
	↓ Secretory Ig A	Prevents pathogen adhesion to the ocular surface epithelia, enhances phagocytosis
	↓ Proline rich 4 protein	Act as an acute phase surfactant to maintain an antimicrobial environment in the ocular surface
	↓ Lactoferrin	Act as an antibacterial, antiangiogenic, antiviral component
	↓ Prolactin-inducible protein	Act as an antibacterial and influences the cell mediated immunity
	↓ Zinc-α-2-glycoprotein	Involved in lipid metabolism
	↓ Proteoglycan 4 (PRG 4/lubricin)	Act as a lubricant and surfactant preventing evaporation
Mucin layer [107,108,109]	↓ MUC5AC, ↓ MUC1, ↓ MUC4, ↓ MUC16	Act as a barrier for pathogens and prevent microbial colonization, maintains the viscosity and surface tension of tear film

↓ decreased.

**Table 2:** Normal function of different components of the OSM, their changes in dry eye, and therapeutic strategies targeting these components.

Components of OSM	Normal Function	Changes in Dry Eye	Targeting Therapy
Cornea	Normal barrier function Growth factors & cytokines Quiescent keratocytes	Scarring & ulcer Opacification Neovascularization Pannus formation Squamous metaplasia Extracellular matrix degradation ↓ Endothelial cell number	Lubricants Autologous serum Growth factors Amniotic membrane extract Amniotic membrane Contact lens MMP-9 inhibitors
Conjunctiva	Immune defense Secrets mucin	Squamous metaplasia ↓ Goblet cell density Chronic inflammation Conjunctivochalasis	Autologous serum Amniotic membrane Vitamin A MMP-9 inhibitor Growth factors Rebamipide Gefarnate Diquafosol tetrasodium Hydroxyeicosatetraenoic acid
Lacrimal Gland	Secretes: Fluid Mucopolysaccharides Electrolyte Microbicidal and other proteins Mucin	↓ Aqueous tear ↓ Acinar and ductal cells Fibrosis Apoptosis Inflammation	Lubricants Immunomodulators Secretagogue Neurostimulation Cyclosporin A
Meibomian Gland	Accomplishes lipogenisis Secretes meibum Maintains tear film stability Prevents tear film evaporation	↓ Meibum ↑ Tear evaporation ↑ Keratinization Apoptosis Inflammation	Warm compress Lid hygiene Lipiflow System™ Intense-Pulsed-Light ω-3 fatty acid Liposomal sprays
Eyelid	Physical defense Meibum distribution Prevents tear film evaporation	↓ Eyelid laxity ↑ Tear evaporation Corneal ulcer Epithelial defect Inflammation Infrequent and ineffective blinking	Warm compress Lid hygiene Antibiotics Surgery
Tear Film	Ocular surface homeostasis Moistens & lubricates Transports nutrient & oxygen	Tear hyperosmolarity Tear film instability Excessive tear film evaporation Delayed tear clearance	Compensation of tear Artificial tears/serum Tear stimulation Pilocarpine, Pituitary adenylate cyclase-activating polypeptide, Diquafosol tetrasodium, Oculeve intranasal neurostimulation device Controlling tear evaporation Moisture-retaining eyeglasses, Swimming goggles, Prosthetic Replacement of the Ocular Surface Ecosystems, Castor oil eye drops Regulating excessive nasolacrimal drainage Punctal plugs
Inflammation	Immune homeostasis	Chronic inflammation ↑ Pro-inflammatory cytokine, ↑ Chemokine ↓ Glandular secretion ↑ Reactive oxygen species ↑ Apoptosis CD4+ T cells-mediated pathogenesis	Corticosteroids Nonsteroidal anti-inflammatory drugs Doxycycline Azithromycin Cyclosporin A FK506 Lifitegrast Tofacitinib Fatty acids
Nerve	Secretes neurotransmitters & nerve growth factors Controls tear reflex & landular secretions	↓ Neuronal stimuli ↓ Corneal sensitivity Altered nerve morphology	Neurostimulation Nerve growth factor
Systemic hormones	Ocular surface homeostasis Regulates meibomain gland & lacrimal gland	Androgen deficiency Estrogen deficiency Thyroid hormones state disorder	Hormonal supplementation Androgen & estrogen receptor inhibitors
Vascular and Lymphatic systems	Transports growth factors Immune response Oxygen supply Lipogenesis	Lymphangiogenesis Hemangiogenesis	Anti lymphangiogenic agents
Ocular surface microbiome	Immune tolerance Eliminates pathogens Mucin turnover	Colonization of normal flora Opportunistic pathogen Drug resistance Infectious keratitis Conjunctivitis	Topical antibiotics Corticosteroids

↓ decreased; ↑ increased.
